# Year in review and appreciation for 2020 reviewers

**DOI:** 10.4069/kjwhn.2020.12.28

**Published:** 2020-12-28

**Authors:** Sue Kim

**Affiliations:** College of Nursing, Yonsei University, Seoul, Korea

What a year it has been, which started as the Year of the Nurse and the Midwife, designated by the World Health Organization, but was swiftly consumed by the coronavirus disease 2019 (COVID-19) pandemic world-wide. Looking back on the *Korean Journal of Women Health* Nursing (KJWHN), we had a dynamic year as well, which I hope to summarize in this editorial, while sharing plans for the upcoming year.

## Year in review and preparing for 2021

This year KJWHN transitioned to a greater number of review articles and an increase in manuscripts available in English. Out of the total number of original manuscripts this year we have published six review papers (including four in this issue), and will continue to welcome integrative reviews, scoping reviews, and systematic reviews and/or meta-analyses that fit our aims and scope. As for increasing English manuscripts in 2020, 13 out of 38 published papers (34.2%; 32 unsolicited, six commissioned manuscripts) and seven out of 32 unsolicited articles (21.9%) were published in English, which will no doubt promote visibility of the important contributions of our authors. KJWHN seeks to steadily increase the proportion of English manuscripts for the benefit of our readers, especially those that offer an international perspective.

This year KJWHN published rich topics in our *Issues and Perspectives* section, starting with the push for nurses to become more familiar with artificial intelligence (AI) in research, education, and practice [1]. COVID-19 has brought limitations in both in-class teaching and clinical practicum, especially for women’s health nursing education. It has also heightened the importance of big data and digital competency. As such, we hope to see more research on how nurses have used digital technology in teaching and are exploring AI application in research and practice.

Another issue paper analyzed the impact of COVID-19 on women and underscored the need for a gendered perspective of data collection in Korea [2]. Since then, an analysis of published COVID-19 research papers have shown that women’s authorship is disproportionately low [3]. Also a study on work status following COVID-19 in Gyeonggido province, Korea, reported how women’s work is being negatively affected while their caregiving burden is increasing [4]. Although gender inequalities in health care is not a new issue, it has certainly emerged with greater importance since COVID-19 [5] and will no doubt challenge us to approach the multiple issues relevant for women with the ongoing pandemic. KJWHN invites commentaries or original manuscripts specifically on COVID-19 impact on women’s health.

In response to our third issue paper’s challenge to nurses and midwives to share their stories as a way to inform the public and promote our profession [6], the Korean Society of Women Health Nursing sent out a call for creative submissions related to nursing and women’s health. Readers can find the essay that won first prize in this issue of KJWHN, and other selected works are posted on the society’s webpage (https://women-health-nursing.or.kr/).

Our last issue paper (December issue) presents a call to action for strengthening midwives as a strategy to alleviate the ultra-low birth rate in Korea. The challenge of Korea’s low birth rate demands serious attention and the Presidential Committee on Ageing Society and Population Policy (PCASPP) has announced the Fourth Framework Plans for Korea on December 15, 2020, which will be the road map for the period 2021 to 2025 [7]. This Framework Plan does not specifically note the potential role of midwives and KJWHN intends to follow up on these developments in 2021, with a paper from PCASPP members and/or key experts.

On another note, author guidelines have been updated as of the December 2020 issue for manuscript length (English manuscripts within 5,000 words, excluding tables, figures, and references) and removing the restriction of overall number of references (although supportive citations in the text should be two or less per argument). These changes have been made to reflect international trends and are available online.

## Appreciation for 2020 reviewers

I am indebted to our reviewers, not to mention our tireless editors and editorial board members, for their commitment and support for KJWHN.

Ahn, Suk Hee (Chungnam National University)

Bae, Kyung Eui (Dongseo University)

Chae, Hyun Ju (Joongbu University)

Chae, Myung-Ock (Cheongju University)

Cheon, Suk Hee (Sangji University)

Cho, In Sook (Inha University)

Cho, Ok Hee (Kongju National University)

Choi, Mi Young (National Evidence-based Healthcare Collaboration Agency)

Choi, So Young (Gyeongsang National University)

Chung, Chae Weon (Seoul National University)

Drake, Emily E. (University of Virginia)

Ha, Ju Young (Pusan National University)

Han, Yong Hee (Hallym Polytechnic University)

Hong, Se Hoon (CHA University)

Hwang, Kyung Hye (Suwon Science College)

Hwang, Moon Sook (Woosuk University)

Jeong, Geum Hee (Hallym University)

Chung, Mi Young (SunMoon University)

Go, Gee Youn (Dongguk University)

Jo, Myung Ju (The Catholic University of Korea)

Kang, Hee Sun (Chung-Ang University)

Kang, Sook Jung (Ewha Womans University)

Kim, Bo Yeoul (Eulji University)

Kim, Hee Kyung (Kongju National University)

Kim, Hee Sook (Dongnam Health University)

Kim, Hyun Kyoung (Kongju National University)

Kim, Jeung Im (Soonchunhyang University)

Kim, Mi Ok (Dankook University)

Kim, Ju Hee (Kyunghee University)

Kim, Kyung Won (Daegue Hanny University)

Kim, Mi Jong (Hannam University)

Kim, Mi Young (Woosuk University)

Kim, Moon Jeong (Pukyung National University)

Kim, Myoung hee (Semyung University)

Kim, Su Hyun (Nambu University)

Kim, Sun Hee (Daegu Catholic University)

Kim, Young Ju (Daegu Health Institute of Technology)

Kim, Yun Mi (Eulji University)

Ko, Eun (Sunchon National University)

Lee, Nae Young (Silla University)

Lee, Kyoung-Eun (Texas A & M University)

Moon, So Hyun (Chosun University)

Nho, Ju Hee (Jeonbuk National University)

Park, Hyun Jung (Pyeongtaek University)

Park, Hye Sook (Dongyang University)

Park, Mi Kyung (Nambu University)

Park, Mee Ra (Changshin University)

Park, Seo A (Gyeongbuk College of Health)

Park, So Mi (Yonsei University)

Shin, Gi Soo (Chung-Ang University)

Son, Hae Kyoung (Eulji University)

Song, Ju Eun (Ajou University)

Sung, Mi Hae (Inje University)

Yeo, Jung Hee (Dong-A University)

Yeom, Gye Jeong (JEI University, Incheon)

Yoon, Ji Won (Shinhan University)

## Journal statistics

Data on manuscripts submitted to KJWHN this year, as of December 19, 2020, are presented in Table 1. While there have been reports of manuscript submissions fluctuating and review processes taking longer with the ongoing COVID-19 [8], KJWHN is fortunate to have had a steady flow of submissions and we thank our authors and society members for their dedication to research and dissemination.

## Upcoming developments

In 2021 KJWHN will offer a series of methods papers on instrument development and validity and reliability, and statistical reports relevant to women’s health in Korea, as collaborative work with Statistics Korea. As noted above, KJWHN will also continue to follow the issues on the ultra-low birth rate in Korea. We hope you will stay tuned for these developments and consider becoming an active part of the dialogue by submitting your scholarly works for publication.

## Figures and Tables

**Table 1.** Basic statistics on manuscripts submitted to the *Korean Journal of Women Health Nursing* in 2020

Category	Data	Notes
Commissioned manuscripts (n)	6	2 Editorials, 4 *Issues & Perspectives*
Unsolicited manuscripts (n)	47	
Accepted manuscripts (n)	32	
Non-accepted manuscripts (n)	13	11 Rejected, 2 withdrawn
Manuscripts reviewed and determined (n)	43	32 Accepted and published, 11 rejected
Manuscripts under review or revision (n)	2	Under revision
Acceptance rate (%)	71.1	32/45=0.711
Average time from submission to acceptance (days)	51.37	
